# Hyperacute changes in blood mRNA expression profiles of rats after middle cerebral artery occlusion: Towards a stroke time signature

**DOI:** 10.1371/journal.pone.0206321

**Published:** 2018-11-15

**Authors:** Marie Dagonnier, William John Wilson, Jenny Margaret Favaloro, Sarah Susan Jane Rewell, Linda Jane Lockett, Stephen Andrew Sastra, Amy Lucienne Jeffreys, Helen Margaret Dewey, Geoffrey Alan Donnan, David William Howells

**Affiliations:** 1 The Florey Institute of Neuroscience and Mental Health, Melbourne Brain Centre, Austin Campus, Heidelberg, Australia; 2 The Commonwealth Scientific and Industrial Research Organisation (CSIRO), Sydney, Australia; 3 School of Medicine, Faculty of Health, University of Tasmania, Hobart, Australia; Fraunhofer Research Institution of Marine Biotechnology, GERMANY

## Abstract

Stroke evolution is a highly dynamic but variable disease which makes clinical decision making difficult. Biomarker discovery programs intended to aid clinical decision making have however largely ignored the rapidity of stroke evolution. We have used gene array technology to determine blood mRNA expression changes over the first day after stroke in rats. Blood samples were collected from 8 male spontaneously hypertensive rats at 0, 1, 2, 3, 6 and 24h post stroke induction by middle cerebral artery occlusion. RNA was extracted from whole blood stabilized in PAXgene tubes and mRNA expression was detected by oligonucleotide Affymetrix microarray. Using a pairwise comparison model, 1932 genes were identified to vary significantly over time (p≤0.5x10^-7^) within 24h after stroke. Some of the top20 most changed genes are already known to be relevant to the ischemic stroke physiopathology (e.g. Il-1R, Nos2, Prok2). Cluster analysis showed multiple stereotyped and time dependent profiles of gene expression. Direction and rate of change of expression for some profiles varied dramatically during these 24h. Profiles with potential clinical utility including hyper acute or acute transient upregulation (with expression peaking from 2 to 6h after stroke and normalisation by 24h) were identified. We found that blood gene expression varies rapidly and stereotypically after stroke in rats. Previous researchers have often missed the optimum time for biomarker measurement. Temporally overlapping profiles have the potential to provide a biological “stroke clock” able to tell the clinician how far an individual stroke has evolved.

## Introduction

Stroke is one of the leading causes of death and of disability [1]. The most specific and biologically powerful treatment for acute ischemic stroke (IS) is thrombolysis with recombinant tissue plasminogen activator (rt-PA) given within the first 4.5 hours of onset [2, 3]. Unfortunately, thrombolysis is disappointingly underused. This is mainly because of uncertainty about diagnosis, time of onset and the perceived risks of cerebral bleeding these entail [4–7]. Even in specialized centers, these limitations mean that fewer than 15% of ischaemic stroke patients can generally be treated with thrombolysis [6, 8, 9].

We already use imaging biomarkers to assist in the clinical decision making process. Indeed, computerized tomography (CT) detection of bleeds is critical for rt-PA use [2, 10]. Penumbral imaging by CT and magnetic resonance imaging (MRI) are increasingly used to select patients likely to respond well to thrombolysis [11, 12]. To date biochemical biomarker studies have focused on confirmation of stroke diagnosis and particularly on differentiation between ischemic and hemorrhagic strokes [13–17]. Whilst some leads are promising none has yet provided a clinically useful biomarker. Lack of specificity and sensitivity limit safe clinical utility and rather surprisingly most measurements have been performed later than the clinically relevant thrombolysis time window [18–20].

Some measurements have been made early in the evolution of a stroke. Cytokine and chemokine expression change rapidly after a range of injuries including stroke [21–23]. Similarly, a number of general and ischemia specific stress response markers such as cortisol, hypoxia-inducible factors (HIFs) and oxidative stress markers such as glutathione S-transferase have been found to change rapidly after stroke [24–28]. However, little attention has been given to the possibility that the pattern of hyperacute changes in gene and protein expression in the blood might provide clinically useful information. This is surprising given the highly dynamic nature of stroke when speed of intervention is known to be critical for good outcome [3, 29, 30].

Systematic reviews have identified over 130 candidate stroke biomarkers selected for investigation primarily because of a known role for the molecule in stroke pathophysiology [18, 31]. However, recent estimates for the human genome have placed the number of genes we possess to be around 19,000 for protein-coding, and 1,500 for non-protein coding [32]. Therefore, stroke researchers have barely scratched the surface of the candidates available.

Because stroke is a highly dynamic but variable process and because previous researchers have largely ignored the rapidity with which changes might take place, we examined the hyperacute responses to stroke through the lens of blood gene-expression over the first 24 hours after stroke in rats. Our explicit goal was to identify profiles of change that could aid clinical decision making. We employed whole genome microarrays to permit an unbiased identification of blood-based RNA expression profiles occurring after stroke. Others have used this technology before [14, 27] but have not focused on the early changes after stroke. Specific acute stroke biomarkers acting as a stroke clock would be valuable in clinical practice to help identify increased numbers of stroke patients who could benefit from thrombolysis.

## Methods

### Ethical approval

This experiment was conducted in accordance with national guidelines (Australian code of practice for the care and use of animals for scientific purposes, 8^th^ edition, 2013). The use of animals (surgical and behavioural procedures) was prospectively approved by the Austin Health Animal Ethics Committee (Austin Health, Heidelberg, Victoria, Australia).

### Animals and induction of stroke

Transient stroke was induced by middle cerebral artery occlusion (MCAo) in eight male 16 week old spontaneously hypertensive rats (SHR). Anesthesia was induced by 5%, and maintained with 2%, isoflurane in a 50:50 O_2_:air mixture through a nose cone. Body temperature was maintained at 37.4°C throughout surgery via a rectal thermocouple and temperature control unit. Laser Doppler flowmetry (moorVMS-LDF, right-angled laser optic probe 0.8mm, MP5b, MoorLab, Devon, UK, coupled to iWORX, 308T, Dover NH, USA) was used to monitor cerebral blood flow (CBF) in the area 5mm lateral and 1mm posterior to bregma, within the MCA perfused cortex. MCAo was achieved using a silicon-coated thread (coating diameter 0.35mm and 2mm length), which was maneuvered through the internal carotid artery approximately 18mm until resistance was felt and a drop in CBF noted on Laser Doppler [33, 34]. All incisions were closed using silk sutures and the animals allowed to recover from anesthesia. Reperfusion under anesthesia was carried out 1.5 hours after vessel occlusion by withdrawing the thread into the external carotid stump and sealing it in place.

Behavioral testing for neurologic deficit was undertaken at 1, 1.5 (immediately prior to anesthesia for reperfusion), 2, 3, 6 and 24 hours post stroke induction using a 5-point scale modified from that of Petullo et al. [35].

At 24 hours post-occlusion, the rats were killed by isoflurane overdose and additional blood (see below) was collected from the heart by syringe. The brain was cut into 2mm slices using a rat brain matrix, and stained with 1% 2,3,5-triphenyltetrazolium chloride (TTC) in saline for 20 minutes (10 minutes each side), before fixation in 10% formalin. Digital images of the stained brain slices were captured on a flatbed scanner (2400dpi), and infarct volume measured using ImageJ. Infarct areas were converted to volumes by finding the average area of two sections and multiplying by the distance between the slices.

### Blood sampling

Animals were warmed briefly under an infrared lamp for approximately 10 minutes to facilitate dilation of the tail veins. Rats were firmly wrapped in a towel and blood collected from the lateral tail vein. Whole blood (250μl) was drawn from the lateral tail vein (1ml syringe, 26G needle) into 500 international units of heparin (500IU, Pfizer) at 0 (immediately before anesthesia induction), 1, 2, 3, and 6 hours post MCA occlusion. The 24 hour sample was taken by heart puncture immediately following anesthetic overdose. After mixing, the samples were centrifuged at 1100g for 10 minutes to separate the plasma and cells. About 150μl plasma was collected onto ice and transferred to -80°C within 15 minutes. Seven hundred microliters of PAXgene solution (collected from PAXgene blood tubes, PreAnalytiX, Qiagen/ BD) was added to the blood cell pellet, the sample mixed and left at room temperature for 10–30 minutes before storage at -80°C until RNA extraction.

### RNA and microarray processing

RNA was prepared from the whole blood cell pellets using the PAXgene Blood RNA Kit (PreAnalytiX #762174, Qiagen). The samples were randomized between 6 batches for workup in groups of 8. The manufacturer’s instructions were followed except in accommodating the 10-fold smaller volume of the initial blood samples, which were in 1.6ml microfuge tubes. The PAXgene pellet was washed in 1 ml of RNase-free water and the final RNA samples were eluted in 40 +20 μl buffer BR5. The RNA was quantitated on a Nanodrop ND-2000 (mean yield 6.9μg) and quality was assessed by the OD_260/280_ ratio (range 1.9–2.2). RNA was stored at -80°C.

Standard Affymetrix protocols were followed for cDNA synthesis, fragmentation and labelling of samples for the microarrays. Briefly, RNA amplification was undertaken with the Nugen Applause WT Amp System (system cat# 5500–24, Millenium Science) using 200ng RNA for the first strand synthesis. cDNA was purified with the Qiagen Min-elute Reaction Clean-up kit and the Encore Biotine module (NuGen) was used for fragmentation and labelling.

Samples were randomly hybridized to the GeneChip RatGene 1.0 ST arrays (Affymetrix, Santa Clara CA, USA; cat#9011730).

### Identification of existing candidate biomarkers

For comparison purposes, a systematic review was performed to identify papers claiming the utility of individual genes as candidate stroke biomarkers because of statistically significant changes reported in the manuscript. Those markers that only showed utility for stroke prognosis or when combined with a panel of other markers were not included in this list.

### Data analysis

Infarct volumes are presented as average ± standard deviation.

For gene expression, pairwise comparisons were made between neighboring time points of the experiment using linear models, and the resulting F-statistic was used to identify genes whose expression varied the most across the time course of the experiment. Models were constructed, and significance testing was performed using the R package *limma* [36]. The level of significance for gene expression change over time was initially set by using the traditional p-value of <0.05. However to reduce false positive discovery this was later set to a p-value of <5x10^-7^. Cluster analysis was performed using a Spearman correlation distance measure followed by a K-means clustering using the R function *kmeans* [36]. Gene expression data was averaged across the samples available for each time point, and mean centered so that similar clusters could be identified across different gene expression magnitudes. Heat maps were constructed using the R package *gplots* [37] and current gene annotations, including homolog identification and array coverage were obtained using the R package *biomaRt* [38].

For comparison of our data with other researchers’ candidate biomarker gene lists, data manipulation and analysis has been conducted in R.

## Results

### Animals

**Fig 1:**
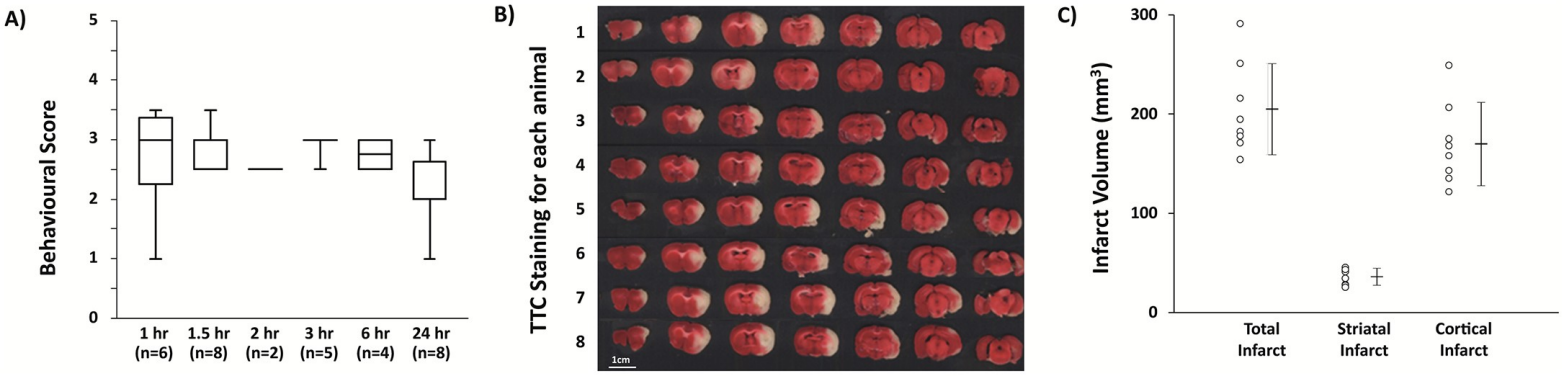
Successful induction of transient MCAo in the SHR. A) Total behavioral deficit score at different time points across the experiment. C) Total, cortical and striatal infarct volumes. Error bars are ± SD. B) TTC stained brain slices showing the extent of ischemic damage at 24 hours post stroke. Circles represent individual animals (1–8), line represents the average measurement.

Stroke surgery was undertaken in 8 SHR rats. All animals were 16 weeks old and weighted 321±13g (range 305-342g) prior to surgery. Anesthesia duration required to induce MCAo was similar across animals (Average 50 minutes; range 41–69 minutes). Stroke was successfully induced in all animals, as evidenced by the development of behavioral deficits and a cerebral infarct at 24 hours post MCAo (Fig 1a and 1c). Average infarct volume was 205.76±45.79mm^3^. Damage consistently involved both the striatum and cortex (36.76±8.29mm^3^, 169.0±41.44mm^3^ respectively; Fig 1b).

### Blood samples

In all 8 rats, the 6 blood samples were collected except for one time point in one rat (6 hours post MCA occlusion in animal 3). A total of 47 samples were therefore collected for biomarker analysis.

### Time responsive genes

**Fig 2:**
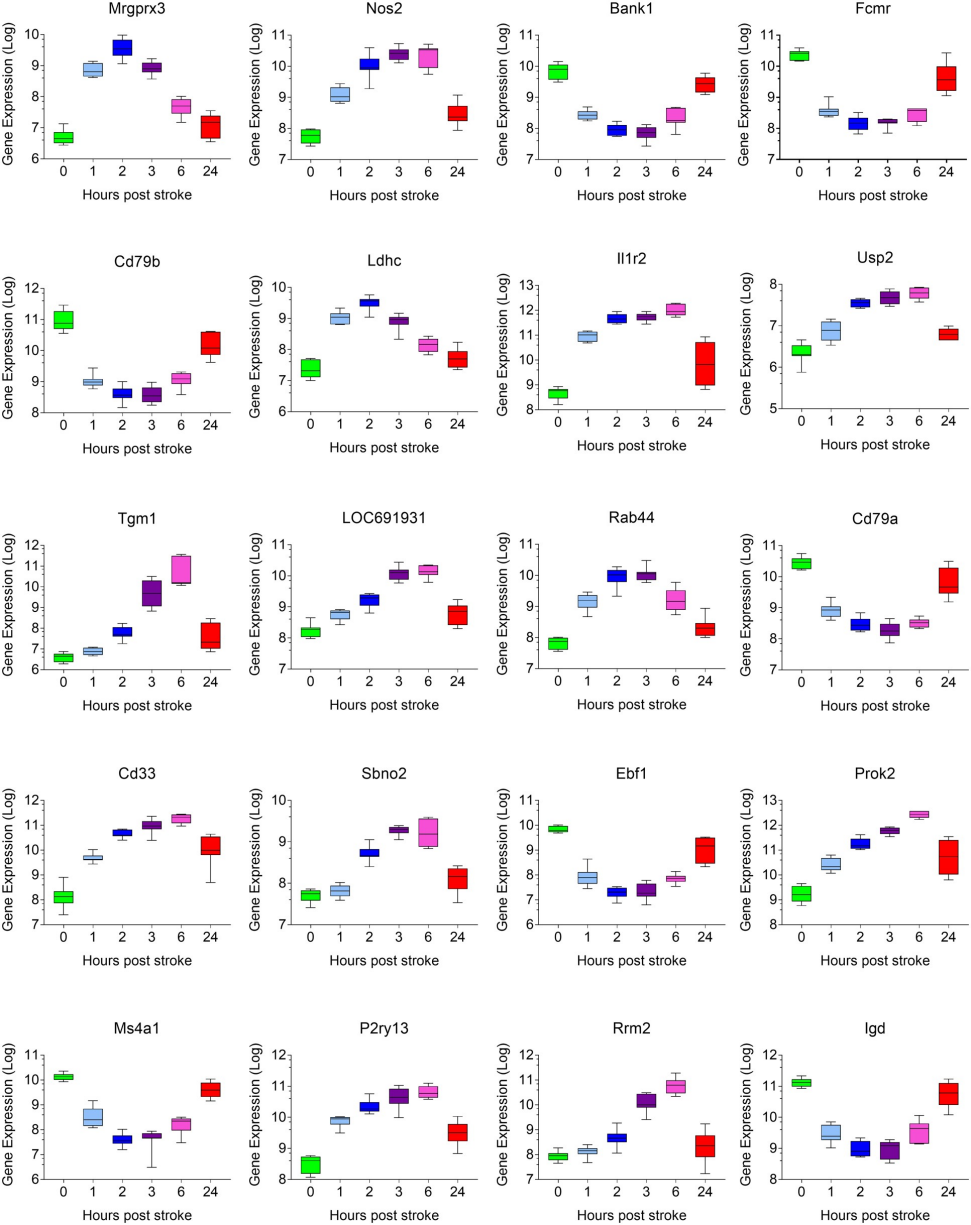
Individual gene expression over time of the 20 most time responsive genes.

Genes with expression varying the most over time were ranked by p-value of the F statistic, with a cut-off of 5x10^-7^. One thousand nine hundred and thirty-two genes reached this level of significance (S1 Table). The 20 most time responsive genes, their functional designation and overall expression are listed in Table 1. Their individual gene expression profiles over time are shown in Fig 2.

**Table 1:** Time responsive genes. Top 20 of the genes with expression varying the most over time (ranked by p-value of the F statistic, p<5x10-7). Genes presented with their p-value, probe ID, and brief gene description.

p-value	Probe ID	Gene Name	Description
1.83E-24	10722208	Mrgprx3	MAS-related GPR, member X3
6.23E-22	10736312	Nos2	nitric oxide synthase 2, inducible
1.5E-21	10826956	Bank1	B-cell scaffold protein with ankyrin repeats 1
3.55E-21	10763768	Fcmr	Fc fragment of IgM receptor
4.77E-21	10748273	Cd79b	Cd79b molecule, immunoglobulin-associated beta
9.45E-21	10707142	Ldhc	lactate dehydrogenase C
1.28E-20	10922816	Il1r2	interleukin 1 receptor, type II
1.39E-20	10909411	Usp2	ubiquitin specific peptidase 2
1.87E-20	10783880	Tgm1	transglutaminase 1
2.46E-20	10826703	LOC691931	hypothetical protein LOC691931
3.08E-20	10828832	Rab44	RAB44, member RAS oncogene family
3.41E-20	10705065	Cd79a	Cd79a molecule-like; Cd79a molecule, immunoglobulin-associated alpha
6.38E-20	10721261	Cd33	CD33 molecule
7.1E-20	10893918	Sbno2	strawberry notch homolog 2 (Drosophila)
7.34E-20	10732941	Ebf1	early B-cell factor 1
7.38E-20	10864433	Prok2	prokineticin 2
8.69E-20	10728883	Ms4a1	membrane-spanning 4-domains, subfamily A, member 1
1.5E-19	10823363	P2ry13	purinergic receptor P2Y, G-protein coupled, 13
1.73E-19	10810144	Rrm2	ribonucleotide reductase M2
1.84E-19	10892472	Igd	immunoglobulin delta heavy chain constant region

### Patterns of gene expression after stroke

**Fig 3:**
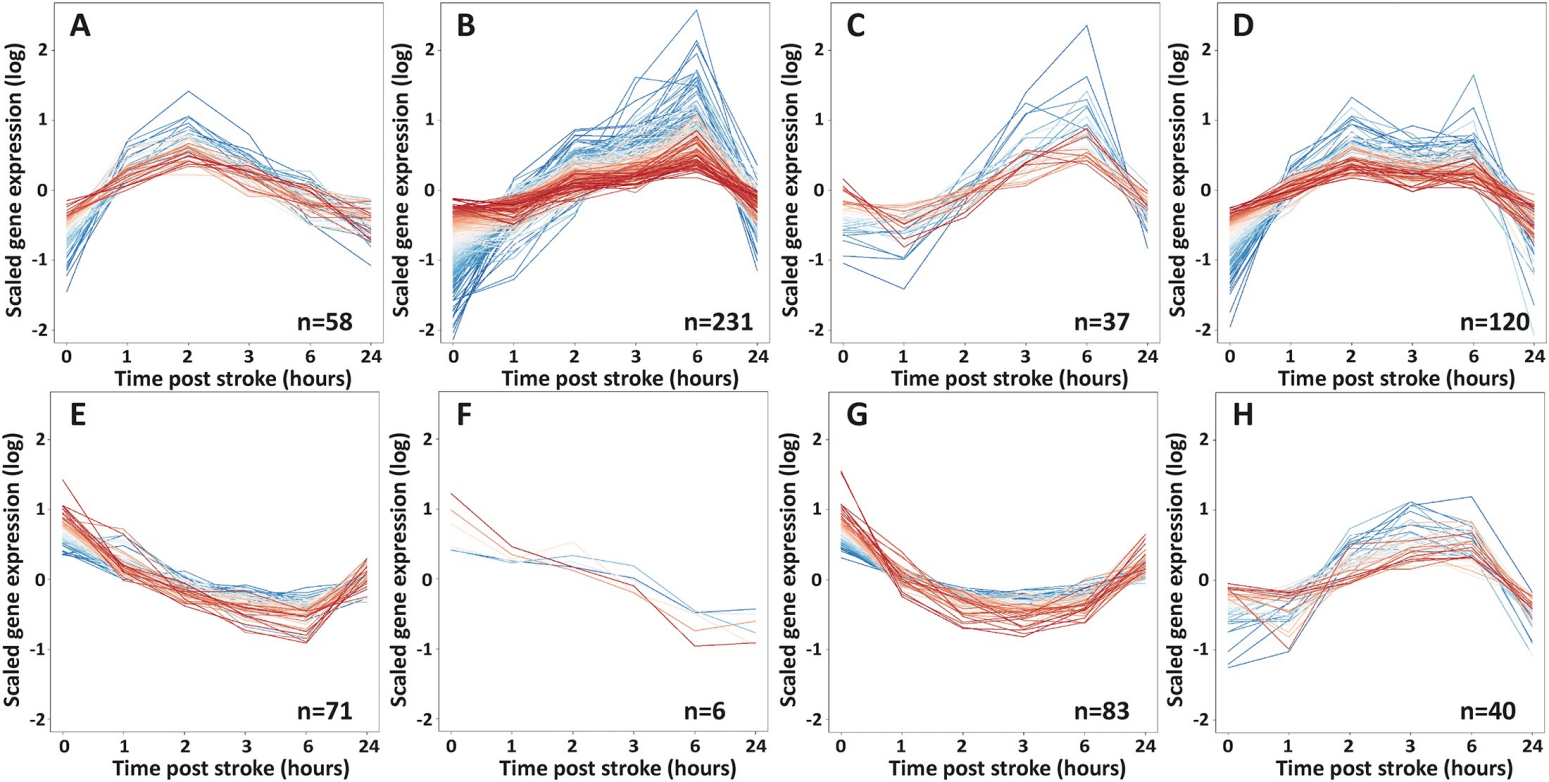
Gene expression clusters. Selected based on their clinically interesting profile. Gene expression values are scaled to the mean expression of 0 and expressed by color (genes highly expressed in red and genes with low expression in blue).

The cluster analysis identified 25 different patterns of gene regulation over time. Eight of them are illustrated here because of their potential clinical utility (Fig 3). Lists of genes with clusters are shown in supplementary data (S2 Table).

These patterns were described as follows:

- Cluster A (58 genes): hyperacute transient upregulation (with expression peaking at 2 hours after stroke with a trend to normalisation by 24 hours)
- Cluster B (231 genes): acute transient upregulation (with expression peaking at 6 hours after stroke then returning to the basal gene expression level by 24 hours
- Cluster C (37 genes): delayed transient upregulation (with unchanging expression for the first hour after stroke then increasing to 6 hours before a return to the basal gene expression level by 24 hours)
- Cluster D (120 genes): 2 peaks of transient upregulation (with a first peak of upregulation by 2 hours after stroke, a decrease of gene expression by 3 hours and a second peak at 6 hours then a return to the basal gene expression level by 24 hours)
- Cluster E (71 genes): progressive transient downregulation (gene expression slowly decreasing up to 6 hours after stroke then starting to return to the basal gene expression level by 24 hours)
- Cluster F (6 genes): progressive downregulation (gene expression slowly decreasing up to 24 hours after stroke)
- Cluster G (83 genes): transient steady downregulation (with expression decreased by 2 hours after stroke and staying to a low plateau up to 6 hours after stroke then starting to return to the basal gene expression level by 24 hours)
- Cluster H (40 genes): early change of regulation (with expression first decreased by 1 hour after stroke then increasing up to 3 or 6 hours after stroke and then returning to the basal gene expression level by 24 hours)

### Comparison with existing candidate biomarkers

Based on the published literature, a list of 73 candidate biomarkers was generated [13, 14, 16, 18, 27, 39–45] and reported in Table 2 using human nomenclature. The majority of this data comprised measurements made far beyond the stroke thrombolysis window. Of the 73 biomarkers listed, one was a protein dimer (Ddimer) and could not be represented by gene expression, and one was a metabolite (homocysteine) which was the result of a metabolic pathway involving several genes. Of the remaining molecules, if the name reported in the literature was different to the current gene annotation, then this was reported in parenthesis, and used to identify matches in our data. To be able to examine their expression levels in our rat data, the corresponding homologous genes were identified in rat if they existed.

**Table 2:** List of ischemic stroke candidate biomarkers based on published results. References: 1 Moore et al. Circulation 2005, 2 Tang et al. JCBFM 2006, 3 Barr et al. Neurology 2010, 4 Grond-Ginsbach et al. J Neurol 2008, 5 Lynch et al. Stroke 2004, 6 Adamski et al. Genomics 2014, 7 Oh et al. J Neuroimmunol 2012, 8 Barr et al. Biol Res Nurs 2014, 9 Lamers et al. Brain Res Bull 2003, 10 Hasan et al. Br J Clin Pharmacol 2012, 11 Laskowitz et al. Stroke 2009, 12 Turck et al. PlosOne 2012.

Molecule (alt name)	Ref	Molecule (alt name)	Ref	Molecule (alt name)	Ref
ACSL1	[7]	F5-1 (F5)	[2]	MMP9	[2],[3],[5],[7],[11]
ADM	[1],[6]	FCGR1A	[1]	NAIP (Naip6 in Rat)	[1]
APLP2	[1]	FOS	[1]	NDKA (NME1)	[12]
ARG1	[2],[3]	FPR1	[2]	NKG7	[7]
BCL6	[2]	FPRL1 (FPR2)	[4]	NPL	[1],[2]
BNP (NPPB)	[11]	GFAP	[9],[10]	NSE (ENO2)	[9]
BST1	[1],[6]	GNLY*	[7]	Nt-proBNP (NPPB)	[12]
C19orf59 (MCEMP1)	[7]	GST-π (GSTP1)	[12]	ORM1	[3]
C3AR1	[4]	HIST2H2AA3	[2]	P selectin (SELP)	[10]
CA4	[2],[3]	homocystein†	[10]	PDE4D	[4]
CCR7	[3]	HOX 1.11 (HOXA2)	[2]	PILRA	[1]
CD14-1	[1]	IL13RA1	[1]	PLBD1	[1],[6]
CD163	[1]	IL18R1	[7]	PYGL	[2],[6]
CD36	[1]	IL18RAP*	[7]	RNASE2	[2]
CD93	[1],[6]	IL1R2	[7]	S100A12	[2],[3],[6]
CKAP4	[2],[6]	IL1RN	[4]	S100A9*	[2],[6]
CRP	[10],[11]	IL6	[12]	S100B	[5],[9]
CSPG2	[3]	IL8*	[8]	S100P	[2]
CYBB	[1],[6]	IQGAP1	[3]	SDPR	[8]
Ddimer‡	[11]	KIAA0146 (SPIDR)	[1]	SLC16A6	[2]
DJ1 (PARK7)	[12]	LOC642103∞	[7]	TLR2	[1]
DUSP1	[1]	LTA4H	[1]	VCAM (Vcam1 in Rat)	[5]
ENTPD1	[1]	LY96*	[2],[3],[8]	VCAN	[1]
EOMES*	[7]	MBP	[9]	VonWillebrand factor (VWF)	[5]
ETS2	[1],[2]	MGAM*	[7]		

*Unavailable on Rat array

^†^A metabolism product, no encoding gene.

^‡^A protein, Fibrin degradation product.

^∞^ discontinued.

**Fig 4:**
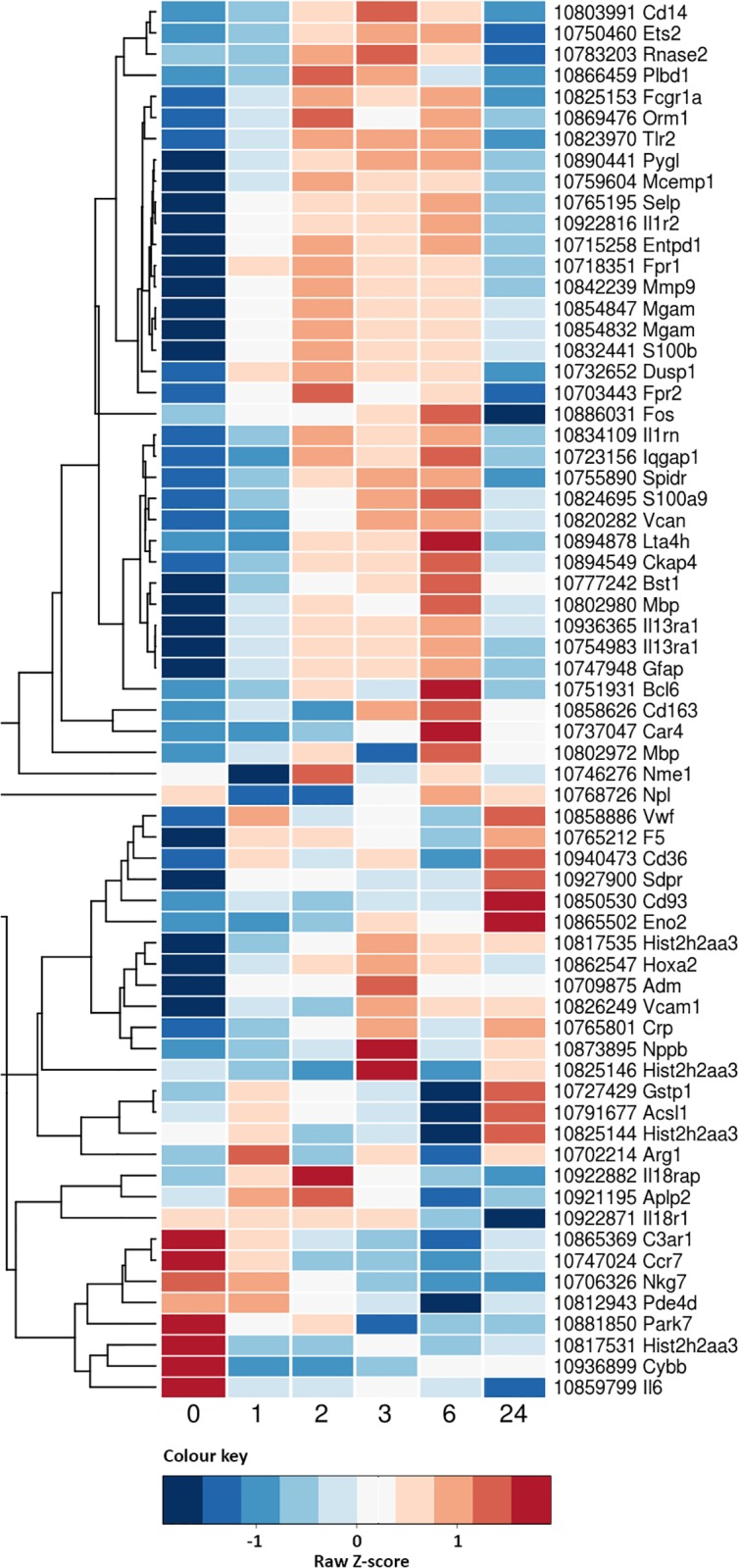
Heat map of the candidate ischemic stroke biomarkers. Genes identified based on published results and their expression in our time course data. Results presented as average values across the 8 animals for a given time point.

We then plotted (as a heat map) the mRNA expression profiles of these published biomarker candidates as detected within our experimental results against time post stroke in rats. This heat map highlighted that, for genes originally selected because of change at 24 hours, expression levels often began to change much earlier (1–3 hours) and indeed often exhibited greater change within this earlier window (Fig 4).

The genes identified as candidate biomarkers from the literature were also identified in our data, ordered by level of significance from our analysis, and compared to our top 1932 time-responsive gene candidates. The ranks showed that 29 of the 59 genes (49%) presented in Table 3 were in our list of most significant changing genes over time, with the highest ranked gene being Interleukin 1 receptor, type II (Il1r2, rank = 8). It should be noted however that most candidates from the literature fall much lower in our ranking of the most time dependently changed genes.

**Table 3:** List of candidate ischemic stroke biomarkers ranked in our data. Genes identified based on published results ranked by p-value of the F statistic based on their gene expression over time in our data set. The red line represents the p<5x10^-7^ cut-off.

Rank	p-value	Probe ID	Gene Name
8	1.28E-20	10922816	Il1r2
141	2.38E-16	10718351	Fpr1
151	4.18E-16	10922882	Il18rap
198	2.89E-15	10765195	Selp
245	8.84E-15	10754983	Il13ra1
257	1.12E-14	10759604	Mcemp1
258	1.13E-14	10820282	Vcan
283	2.92E-14	10824695	S100a9
288	3.17E-14	10842239	Mmp9
298	4.35E-14	10750460	Ets2
334	1.4E-13	10823970	Tlr2
381	3.4E-13	10737047	Car4
406	5.93E-13	10715258	Entpd1
445	1.24E-12	10890441	Pygl
538	7.39E-12	10825153	Fcgr1a
641	4.43E-11	10777242	Bst1
652	4.7E-11	10803991	Cd14
667	5.67E-11	10751931	Bcl6
746	1.35E-10	10747024	Ccr7
850	4.08E-10	10894549	Ckap4
998	1.28E-09	10834109	Il1rn
1140	4.12E-09	10783203	Rnase2
1337	2.07E-08	10732652	Dusp1
1385	2.79E-08	10723156	Iqgap1
1414	3.19E-08	10865369	C3ar1
1533	5.96E-08	10703443	Fpr2; Fpr2l
1767	2.18E-07	10894878	Lta4h
1799	2.67E-07	10761025	Pilra
1925	4.86E-07	10869476	Orm1
2033	7.88E-07	10791677	Acsl1
2124	1.23E-06	10850530	Cd93
2264	1.96E-06	10866459	Plbd1
2358	2.59E-06	10854733	Mgam
2678	0.000007	10812943	Pde4d
2686	0.000007	10927900	Sdpr
3647	0.0000592	10739323	Slc16a6
3981	0.0001	10765801	Crp
4508	0.0003	10768726	Npl
4834	0.0004	10755890	Spidr
5928	0.0012	10747948	Gfap
6811	0.0027	10826249	Vcam1
7018	0.0032	10858626	Cd163
7129	0.0035	10706326	Nkg7
8997	0.0116	10858886	Vwf
9093	0.0121	10886031	Fos
9599	0.0154	10881850	Park7
9657	0.0158	10832441	S100b
10160	0.0201	10765212	F5
10969	0.0276	10936899	Cybb
~	NS	10709875	Adm
~	NS	10802980	Mbp
~	NS	10873895	Nppb
~	NS	10859799	Il6
~	NS	10922871	Il18r1
~	NS	10921195	Aplp2
~	NS	10702214	Arg1
~	NS	10865502	Eno2
~	NS	10727429	Gstp1
~	NS	10746276	Nme1

### Effect of surgery and anesthesia

To explore the difficulty of selecting appropriate controls in animal experiments requiring surgery to induce stroke, and which our experiment lacked, we also compared our data set with an available list of genes potentially specific for the impact of surgery and anesthesia. In 2001, Tang et al. studied the blood gene expression for different neurological conditions including stroke. They identified genes significantly (more than two fold) over or under expressed in the ischemic stroke and sham groups after 24 hours compared to untouched controls [46]. Twenty five genes were shown to be significantly over expressed in the animal blood at 24 hours after ischemic stroke while 98 had significantly decreased expression. In the sham group, 40 genes were upregulated and 126 downregulated at 24 hours after the intervention. These four lists of up- and downregulated genes for ischemic stroke and sham models were obtained from Tang et al. supplementary data. Their ‘genes’ were reported as probesets from the Rat U34A array.

To determine whether the up and downregulated specific stroke genes identified by Tang were present in our dataset and exhibited a similar response, gene names were mapped between the two datasets. For this, Tang’s probesets were first annotated with current gene designations from the rat genome using the R library *biomaRt* [38]. This library was also used for mappings between the Affymetrix GeneChip Rat Gene 1.0st platform used for our data collection, and the older U34A which Tang employed.

**Fig 5:**
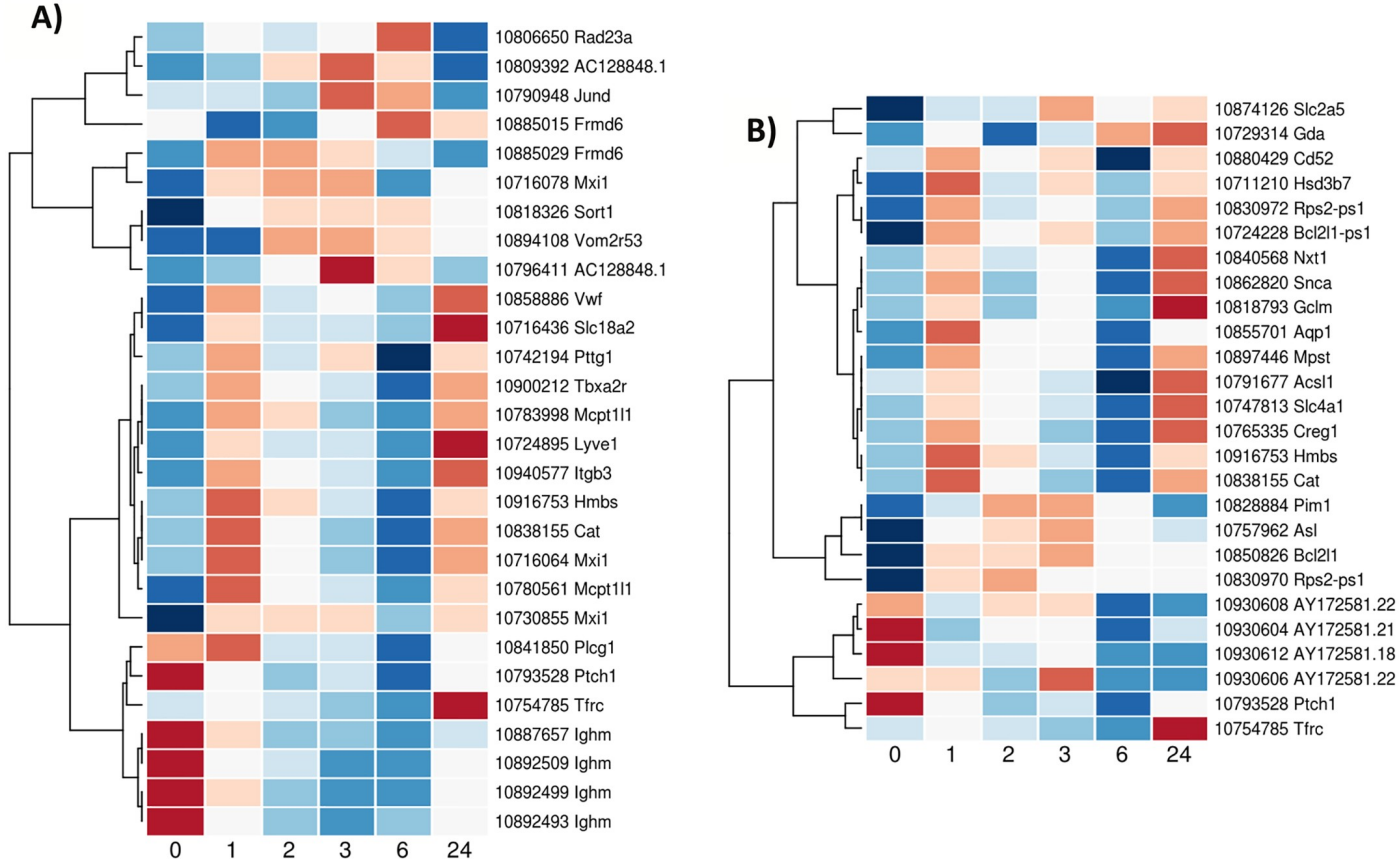
Heat maps of the Tang’s A) upregulated ischemic stroke genes and B) upregulated Sham genes. Genes identified by Tang et al. and their expression in our time course data. Results presented as average values across the 8 animals for a given time point and colour key is similar to the one used in Fig 4.

**Fig 6:**
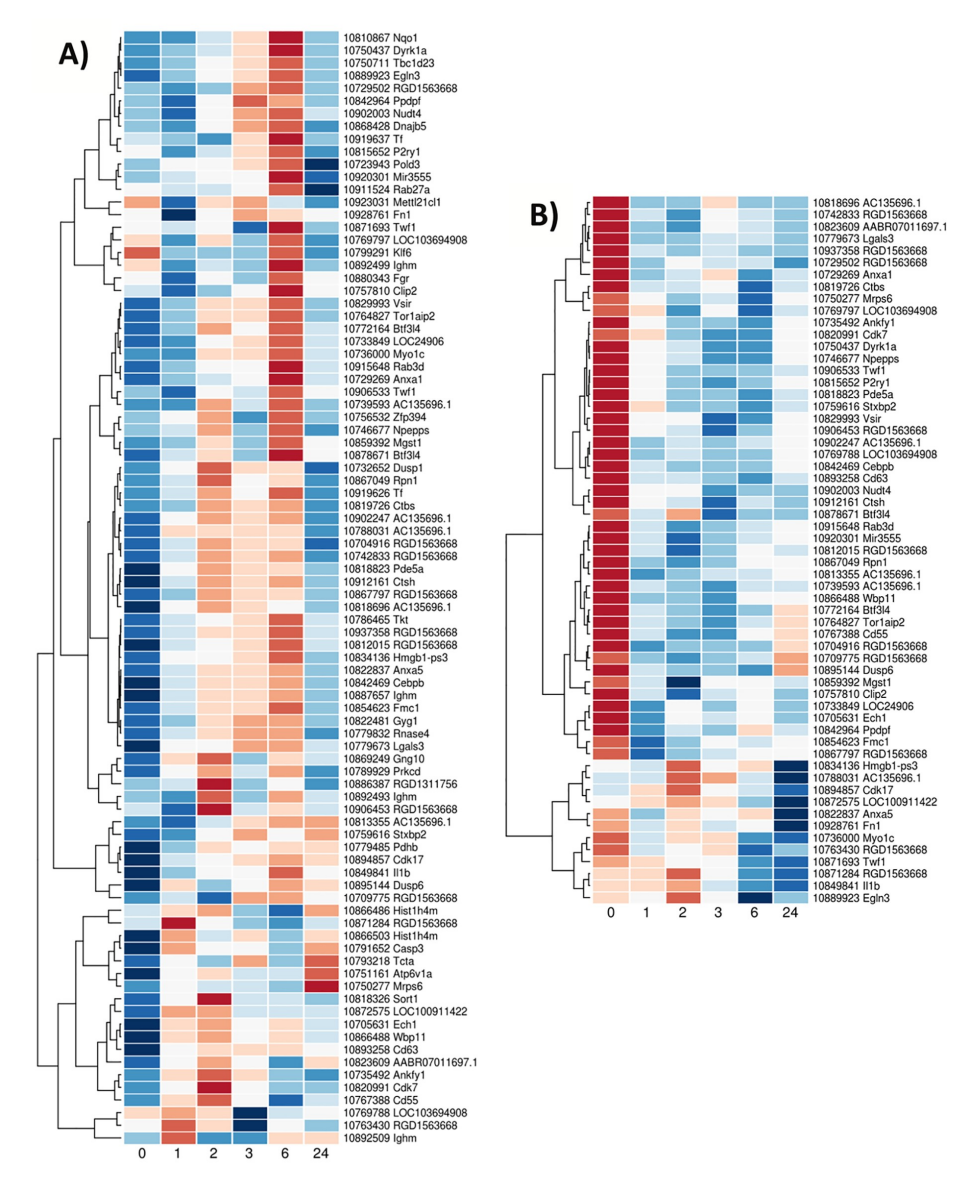
Heat map of the Tang’s downregulated Sham genes. Genes identified by Tang et al. and their expression in our time course data. Figure separated in A and B for better image quality. Results presented as average values across the 8 animals for a given time point and colour key is similar to the one used in Fig 4.

**Fig 7:**
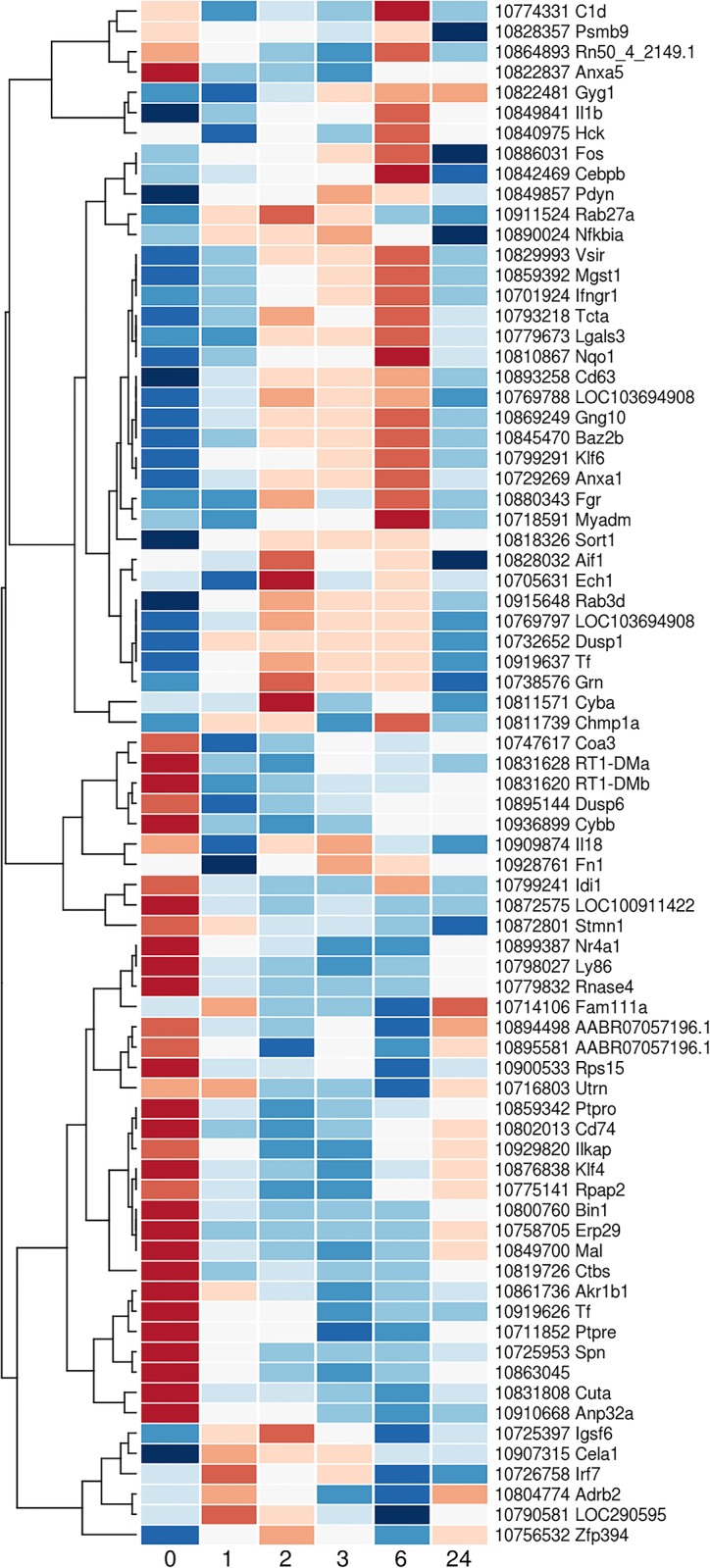
Heat map of the Tang’s downregulated ischemic stroke genes. Genes identified by Tang et al. and their expression in our time course data. Results presented as average values across the 8 animals for a given time point and colour key is similar to the one used in Fig 4.

The sham and stroke up- and downregulated genes identified by Tang were extracted from our stroke data and plotted in 4 different heat maps (Figs 5 and 6). These 4 heat maps confirmed that expression of these genes varied over time after stroke and that some of these changes occurred very early after stroke (as early as after one hour post vessel occlusion). In the significantly downregulated genes after stroke, more than a third of the genes identified by Tang et al. were actually first upregulated between 2 and 6 hours after MCA occlusion and only then downregulated at 24 hours (top half of Fig 7). This subset of downregulated genes after stroke contrasts with another subset also represented in the same heat map where the genes appeared to be upregulated before the start of the surgery then downregulated over a 24 hour period after stroke, with minimal expression observed between 2 and 6 hours after stroke (bottom half of Fig 7). Interestingly, from the heat map plot of genes from Tang’s sham downregulated class (Fig 6 separated in A and B for better image quality), we can identify a subset of genes whose expression is suppressed after the initiation of the anesthesia and start of the surgery and with a maximum of downregulation peaking for the samples collected at 2 to 6 hours (after the end of the anesthesia period, Fig 6B).

Focusing on the 24 hour time point, it appeared that not all the genes identified as up or downregulated by Tang showed these characteristics in our dataset. The concordance between genes identified as downregulated by Tang et al. and effectively downregulated in our dataset was better than for the upregulated genes.

**Fig 8:**
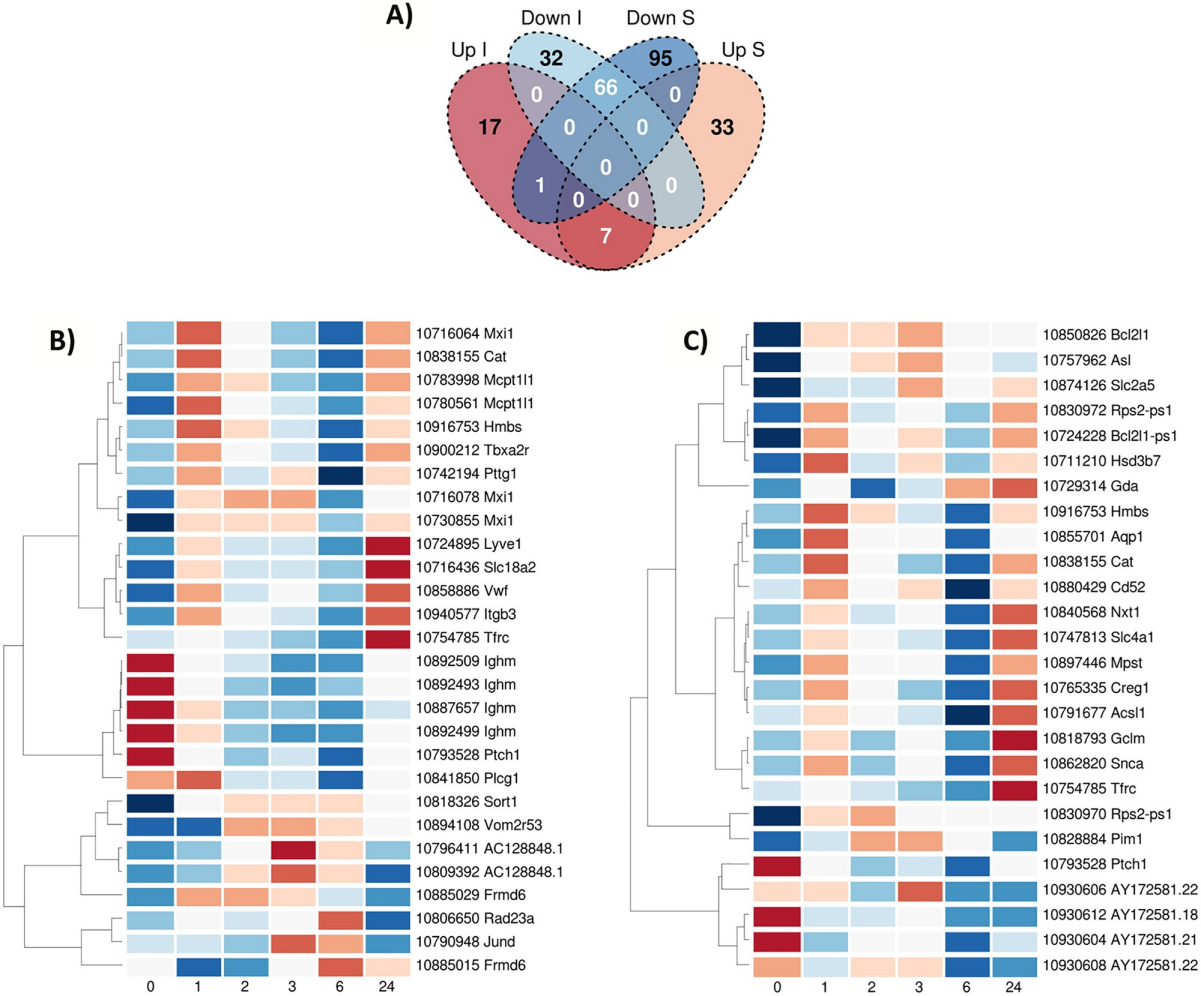
Analysis of the gene lists published by Tang for condition specificity. A) Direct comparison of the up and downregulated genes for the ischemic stroke and the Sham groups and creation of a Venn diagram. B) Heat map of the upregulated specific ischemic stroke genes. C) Heat map of the downregulated specific ischemic stroke genes. Genes identified by the analysis of Tang’s results and their expression in our time course data. Results presented as average values across the 8 animals for a given time point and colour key is similar to the one used in Fig 4.

The gene lists published by Tang et al. were also reanalyzed for condition specificity. We performed a direct comparison of the up and downregulated genes for the ischemic stroke and the sham groups and generated a Venn diagram of the significantly differently expressed specific genes for the two conditions (Fig 8A). We identified 49 genes whose expression was significantly changed only in the ischemic stroke group (17 upregulated and 32 downregulated genes).

These potential ischemic-specific genes were then ranked in our prioritized list of the top 1932 genes varying over time after stroke (ordered by the p-value of the F-statistic). From the 17 genes upregulated exclusively in the Tang stroke group, 16 were successfully matched in our data set and four of them were found to be in our top 1932 gene list (Table 4). From the downregulated list of 32 genes, 24 were successfully matched and 7 were present in our top list (Table 5). The expression levels of these potential specific ischemic genes were reanalyzed over time in our rat data set and the corresponding heat maps generated (Fig 8B and 8C). These heat maps also demonstrated a time evolving pattern of expression after stroke which was not as well defined as that for the other candidate biomarkers identified from the literature (Table 2).

**Table 4:** List of upregulated potential specific ischemic stroke genes ranked in our data. Genes identified by Tang et al. ranked by p-value of the F statistic based on their gene expression over time in our data set. The red line represents the p<5x10-7 cut-off.

Rank	p-value	Probe ID	Gene Name
182	1.99E-15	10818326	Sort1
1049	1.99E-09	10809392	Mt1a
1754	1.96E-07	10841850	Plcg1
1994	6.67E-07	10724895	Lyve1
2703	7.25E-06	10730855	Mxi1
4685	0.0003	10831567	RT1-Bb
6406	0.0019	10806650	Rad23a
6787	0.0027	10716436	Slc18a2
8839	0.0106	10940577	Itgb3
8997	0.0116	10858886	Vwf
10727	0.0252	10790948	Jund
12554	0.0496	10783998	Mcpt1
~	NS	10742194	Pttg1
~	NS	10900212	Tbxa2r
~	NS	10885015	Frmd6
~	NS	10894108	Vom2r53

**Table 5:** List of downregulated potential specific ischemic stroke genes ranked in our data. Genes identified by Tang et al. ranked by p-value of the F statistic based on their gene expression over time in our data set. The bold line represents the p<5x10^-7^ cut-off.

Rank	p-value	Probe ID	Gene Name
291	3.59E-14	10802013	Cd74
683	6.75E-11	10798027	Ly86
727	1.11E-10	10718591	Myadm
869	4.66E-10	10895581	LOC689014
939	7.82E-10	10899387	Nr4a1
1277	1.27E-08	10861736	Akr1b1
1875	4.00E-07	10800760	Bin1
2110	1.16E-06	10711852	Ptpre
2664	6.56E-06	10910668	Anp32a
2899	1.18E-05	10716803	Utrn
3352	3.25E-05	10828357	Psmb9
3824	8.22E-05	10799241	Idi1
5939	0.0013	10890024	Nfkbia
6069	0.0014	10758705	Erp29
6248	0.0017	10726758	Irf7
7758	0.0056	10714106	Fam111a
9161	0.0125	10907315	Cela1
9823	0.0173	10790581	LOC290595
10478	0.0228	10775141	Rpap2
~	NS	10863045	AABR07051563.1
~	NS	10864893	Akr1b1-ps2
~	NS	10929820	Ilkap
~	NS	10804774	Adrb2
~	NS	10811739	Chmp1a

## Discussion

The use of tPA therapy in ischemic stroke is largely limited by uncertainty about the time of stroke onset. The main objective of this study was to provide proof of principle that changes in gene expression profiles after stroke might provide a time dependent signature. The current study clearly identified significant hyperacute changes in the patterns of mRNA expression in the blood of rats over time after MCAo, and that the elaboration of a stroke clock based on gene expression patterns from blood is indeed feasible. Such a stroke clock would allow clinicians to understand where in the highly dynamic evolution of a stroke a patient is when they first present at hospital. Specific blood biomarkers capable of predicting stroke onset time may expedite stroke diagnosis in the emergency department and help increasing the number of patients eligible for intravenous thrombolysis or other beneficial intravascular procedures.

To identify the most variable genes over time after stroke, we used a pairwise comparison model and significance testing. Our initial analysis found 13000 genes significantly changing over time with the classical p-value cut off for significance of 0.05. Since this would still give 5% false positives (equivalent to more than 1300 genes in our data set), we decided to increase the level of significance required to a p-value of 5x10^-7^ to reduce false discovery.

Despite the lack of control group in our experiment, the biology of the genes identified by this highly selective regime as the most variable over time is consistent with what we known of the IS physiopathology. Some of the top 20 most time responsive genes appear to be genes that are relevant to the biology of the injury. For example, the interleukin-1 receptor (Il-1R) has been a target for antagonism in the management of acute stroke. Blockage of Il-1 action via its receptors has been shown to reduce brain damage in rodent model of cerebral ischemia [47, 48]. It is also well known that the neuronal nitric-oxide synthase plays a crucial role in the regulation of cerebral blood flow against pathogenic factors associated with cerebral ischemia [49]. Prokineticin 2 (Prok2) has also recently been identified as a deleterious mediator for cerebral ischemia [50].

By cluster analysis, we found evidence for more different patterns of gene expression than expected. The majority of genes showed first either an upregulation or downregulation immediately after the ischemic event and then a progressive trend to return to their basal activity at 24 hours. These included gene expression profiles with high potential clinical utility including hyperacute or acute transient upregulation/downregulation (with broad or very narrow expression peaks (or troughs) at different points within the 2 to 6 hour window after stroke (Fig 3A and 3B). Many genes showed complex expression profiles with more than one peak of expression (Fig 3D) or varied in opposite directions (Fig 3C and 3H) over time.

In our experiment, the shape of the profiles for different gene sets identified differs dramatically and will allow the construction of a stroke clock where the expression patterns of a series of genes would be able to define the evolution of the stroke damage. Indeed, by pulling out the gene expression levels of Mrgprx3, Prok2 and Bank1 (all part of the top 20 most variable genes over time list) and combining them 2 by 2 at 3 different time points (expression of Bank1 and Mrgprx3 at 2 hours, expression of Bank1 and Prok2 at 6 hours and expression of Bank1 and Mrgprx3 at 24 hours; see striking image), we could create a tool capable of determining the stroke onset time.

The strength of our study is the use of sequential blood drawing in the same animals and the high frequency of repeated blood samplings starting hyperacutely after the initiation of the ischemic brain injury. Our finding gives a new perspective on most previously published stroke biomarker results. By drawing blood samples repetitively in the first 24 hours after stroke, we have been able to provide new evidence that many candidate biomarkers change hyperacutely after stroke and trend back towards their basal value within 24 hours.

Importantly, our study demonstrated that our identified biomarkers profiles are highly reproducible across individual animals and that changes in gene expression are of a significant magnitude (multiple log change). These observations are consistent with highly reproducible induction of stroke and collection of blood at well-defined times after stroke highlighting the utility of animal models in the quest for ischemic stroke biomarkers.

By analyzing the expression of Tang’s gene lists (based on samples collected at 24 hours after stroke induction) within our data set, we found that marked changes of gene expression had occurred within the first 24 hours after stroke. We also noted differences in the identification of up or downregulated expression of some genes at 24 hours after stroke or sham surgery. Finally, we found that only a small proportion of the genes presented by Tang as potentially specific for ischemic stroke were part of our list of time responsive genes (when ranked by the p-value of the F-statistic and with a p-value cut off of 5x10^-7^). It is not too surprising that our results differed from Tang’s. Firstly, arrays have evolved considerably over the last ten years. Tang’s expression data were collected on the Affymetrix GeneChip Rat Genome U34A array which contained probesets for 7,000 full-length sequences, and about 1,000 EST clusters. The Affymetrix GeneChip RatGene 1.0 ST array offers a coverage of more than 27,000 protein coding transcripts and 24,000 Entrez genes. Our data set is therefore enriched. This also explains why we could not find a match in our data set for all the genes presented by Tang. Mapping of older Affymetrix UTR-probeset array technology to the current rat genome was not perfect, as the Rat genome has been refined and improved since the U34A array was designed. As a result not all the genes, or probesets, identified from analysis of the array data had current annotations in the rat genome and in some cases U34A probesets matched to multiple genes. Differences observed between the two experiments at the 24 hours sampling time can also be explained by a difference of collecting methods (Tang et al. did not used PAXgene tubes and mixed the blood from two different animals to have sufficient blood to conduct the microarrays). The use of a single time point of 24 hours can also account for the relative short list of upregulated genes specific to stroke that Tang presented since we find that most genes have returned to near normal by this late time.

The reanalysis of the other genes identified as candidate stroke biomarkers from the human literature showed a greater overlap with our data. More than half of these genes were present in our most significant time responsive gene list. This argues in favor of the genes present in our list being specific to stroke physiopathology and that the changes in gene expression observed in our data are unlikely to all be due to the surgery or anesthesia required to induce stroke in our model system since this is not a feature of the human studies. This concordance of the rat and human data points to the potential for successful translation. It should be noted however that others have cautioned on extrapolating from rat to human data [19]. The heat maps created with the literature gene lists presented time patterns similar to those we identified with our cluster analysis. Even if these candidate biomarker genes were initially not identified for this use, they have the potential to contribute to a time signature for stroke. The more dramatic time signatures discovered in our unbiased whole genome experiment suggest that many of these genes may have even greater utility for this purpose.

Candidate biomarkers have rarely been selected in the context of looking for a stroke clock. Turck et al. analyzed blood samples taken at 2 different time points (within 3 hours and within 36 hours) after stroke but had to combine two separate cohorts of patients to achieve their aim [27]. The only study collecting sequential human blood samples in the same individual early after stroke was conducted by Tang et al. in 2006 but their attention focused on elaboration of a diagnostic tool capable of differentiating ischemic stroke patients from controls and the earliest blood samples were collected 3 hours after the ischemic event [14]. Therefore, this study is to our knowledge the first to combine whole genome screening and sequential blood sampling within a time frame capable of informing clinical decision making.

All experiments have limitations. Our study will need to be replicated and to include a comprehensive series of sham groups to provide information about the specificity of these biomarkers to brain ischemia and the potentially cofounding roles of surgery, ischemia in other tissues (primarily neck and jaw), anesthesia and stress. The experiment will also have to be replicated in female rats to account for sexual dysmorphism in stroke. Finally, the data reported here comes from an animal model of stroke. Such data can only be hypothesis generating and the changes identified here will need to be replicated in studies of humans with stroke. Nevertheless, in mass spectrometry analysis of plasma from patients with stroke, we have already found that protein expression in humans displays similar temporal profiles [51].

In conclusion, we have demonstrated that gene expression changes over time after ischemic stroke and that this change occurs quickly after the event. Gene expression might be used to construct a “Stroke Clock” which provides important information on patient selection for acute stroke therapy.

## Supporting information

S1 Table Time responsive genes. List of the 1932 the genes with expression significantly varying over time (ranked by p-value of the F statistic, p<5x10^-7^). Genes presented with their p-value, probe ID, Ensemble gene ID and brief gene description.
(XLSX)

S2 Table Clusters composition. Lists of genes composing each individual cluster.
(XLSX)

## References

[1] Roger VL, Go AS, Lloyd-Jones DM, Benjamin EJ, Berry JD, Borden WB et al Executive summary: heart disease and stroke statistics—2012 update: a report from the American Heart Association. Circulation. 2012;125(1):188–97. 10.1161/CIR.0b013e3182456d46 22215894 10.1161/CIR.0b013e3182456d46

[2] Hacke W, Kaste M, Bluhmki E, Brozman M, Dávalos A, Guidetti D et al Thrombolysis with Alteplase 3 to 4.5 Hours after acute ischemic stroke. N Engl J Med. 2008;359(13):1317–29. 10.1056/NEJMoa0804656 18815396 10.1056/NEJMoa0804656

[3] Jauch EC, Saver JL, Adams HP, Bruno A, Connors JJ, Demaerschalk BM et al Guidelines for the early management of patients with acute ischemic stroke. A Guideline for healthcare professionals from the American Heart Association/American Stroke Association. Stroke; a journal of cerebral circulation. 2013;44(3):870–947. 10.1161/STR.0b013e318284056a 23370205

[4] Barber PA, Zhang J, Demchuk AM, Hill MD, Buchan AM. Why are stroke patients excluded from TPA therapy? An analysis of patient eligibility. Neurology. 2001;56(8):1015–20. 11320171 10.1212/wnl.56.8.1015

[5] Fink JN, Kumar S, Horkan C, Linfante I, Selim MH, Caplan LR et al The stroke patient who woke up. Clinical and radiological features, including diffusion and perfusion MRI. Stroke; a journal of cerebral circulation. 2002;33(4):988–93. 10.1161/01.str.0000014585.17714.67 11935049

[6] Faiz KW, Sundseth A, Thommessen B, Ronning OM. Reasons for low thrombolysis rate in a Norwegian ischemic stroke population. Neurological sciences: official journal of the Italian Neurological Society and of the Italian Society of Clinical Neurophysiology. 2014;35(12):1977–82. 10.1007/s10072-014-1876-4 25030125 10.1007/s10072-014-1876-4

[7] Albers GW, Amarenco P, Easton JD, Sacco RL, Teal P. Antithrombotic and thrombolytic therapy for ischemic stroke: American College of Chest Physicians Evidence-Based Clinical Practice Guidelines (8th Edition). Chest. 2008;133(6 Suppl):630S–69S. 10.1378/chest.08-0720 18574275 10.1378/chest.08-0720

[8] Minnerup J, Wersching H, Ringelstein EB, Schilling M, Schäbitz WR, Wellmann J et al Impact of the extended thrombolysis time window on the proportion of recombinant tissue-type plasminogen activator-treated stroke patients and on door-to-needle time. Stroke; a journal of cerebral circulation. 2011;42(10):2838–43. 10.1161/STROKEAHA.111.616565 21852612

[9] Krogias C, Bartig D, Kitzrow M, Weber R, Eyding J. Trends of hospitalized acute stroke care in Germany from clinical trials to bedside. Comparison of nation-wide administrative data 2008–2012. Journal of the neurological sciences. 2014;345(1–2):202–8. 10.1016/j.jns.2014.07.048 25109534 10.1016/j.jns.2014.07.048

[10] The National Institute of Neurological Disorders and Stroke rt-PA Stroke Study Group. Tissue plasminogen activator for acute ischemic stroke. N Engl J Med. 1995;333(24):1581–7. 10.1056/NEJM199512143332401 7477192 10.1056/NEJM199512143332401

[11] Davis S, Donnan GA. Time is Penumbra: imaging, selection and outcome. The Johann jacob wepfer award 2014. Cerebrovasc Dis. 2014;38(1):59–72. 10.1159/000365503 25227260 10.1159/000365503

[12] Wintermark M, Luby M, Bornstein NM, Demchuk A, Fiehler J, Kudo K et al International survey of acute Stroke imaging used to make revascularization treatment decisions. International journal of stroke: official journal of the International Stroke Society. 2015;10(5):759–62. 10.1111/ijs.12491 25833105 10.1111/ijs.12491 PMC5286907

[13] Moore DF, Li H, Jeffries N, Wright V, Cooper RA, Jr., Elkahloun A et al Using peripheral blood mononuclear cells to determine a gene expression profile of acute ischemic stroke: a pilot investigation. Circulation. 2005;111(2):212–21. 10.1161/01.CIR.0000152105.79665.C6 15630028 10.1161/01.CIR.0000152105.79665.C6

[14] Tang Y, Xu H, Du X, Lit L, Walker W, Lu A et al Gene expression in blood changes rapidly in neutrophils and monocytes after ischemic stroke in humans: a microarray study. Journal of cerebral blood flow and metabolism: official journal of the International Society of Cerebral Blood Flow and Metabolism. 2006;26(8):1089–102. 10.1038/sj.jcbfm.9600264 16395289 10.1038/sj.jcbfm.9600264

[15] Stamova B, Xu H, Jickling G, Bushnell C, Tian Y, Ander BP et al Gene expression profiling of blood for the prediction of ischemic stroke. Stroke; a journal of cerebral circulation. 2010;41(10):2171–7. 10.1161/STROKEAHA.110.588335 20798371 10.1161/STROKEAHA.110.588335 PMC2987675

[16] Barr TL, Conley Y, Ding J, Dillman A, Warach S, Singleton A et al Genomic biomarkers and cellular pathways of ischemic stroke by RNA gene expression profiling. Neurology. 2010;75(11):1009–14. 10.1212/WNL.0b013e3181f2b37f 20837969 10.1212/WNL.0b013e3181f2b37f PMC2942033

[17] Dvorak F, Haberer I, Sitzer M, Foerch C. Characterisation of the diagnostic window of serum glial fibrillary acidic protein for the differentiation of intracerebral haemorrhage and ischaemic stroke. Cerebrovasc Dis. 2009;27(1):37–41. 10.1159/000172632 19018136 10.1159/000172632

[18] Hasan N, McColgan P, Bentley P, Edwards RJ, Sharma P. Towards the identification of blood biomarkers for acute stroke in humans: a comprehensive systematic review. British journal of clinical pharmacology. 2012;74(2):230–40. 10.1111/j.1365-2125.2012.04212.x 22320313 10.1111/j.1365-2125.2012.04212.x PMC3630743

[19] Sharp FR, Jickling GC. Whole genome expression of cellular response to stroke. Stroke; a journal of cerebral circulation. 2013;44(6 Suppl 1):S23–5. 10.1161/STROKEAHA.112.679357 23709718 10.1161/STROKEAHA.112.679357 PMC3888808

[20] Whiteley W, Chong WL, Sengupta A, Sandercock P. Blood markers for the prognosis of ischemic stroke: a systematic review. Stroke; a journal of cerebral circulation. 2009;40(5):e380–9. 10.1161/STROKEAHA.108.528752 19286602 10.1161/STROKEAHA.108.528752

[21] Wang Q, Tang XN, Yenari MA. The inflammatory response in stroke. Journal of neuroimmunology. 2007;184(1–2):53–68. 10.1016/j.jneuroim.2006.11.014 17188755 10.1016/j.jneuroim.2006.11.014 PMC1868538

[22] Nilupul Perera M, Ma HK, Arakawa S, Howells DW, Markus R, Rowe CC et al Inflammation following stroke. Journal of clinical neuroscience: official journal of the Neurosurgical Society of Australasia. 2006;13(1):1–8. 10.1016/j.jocn.2005.07.005 16410192 10.1016/j.jocn.2005.07.005

[23] Clark W, Lutsep H. Potential of anticytokine therapies in central nervous system ischaemia. Expert Opin Biol Ther 2001;1(2):227–37. 10.1517/14712598.1.2.227 11727532 10.1517/14712598.1.2.227

[24] Zi WJ, Shuai J. Cortisol as a prognostic marker of short-term outcome in chinese patients with acute ischemic stroke. PloS one. 2013;8(9):e72758 10.1371/journal.pone.0072758 24069157 10.1371/journal.pone.0072758 PMC3771965

[25] Barugh AJ, Gray P, Shenkin SD, MacLullich AM, Mead GE. Cortisol levels and the severity and outcomes of acute stroke: a systematic review. Journal of neurology. 2014;261(3):533–45. 10.1007/s00415-013-7231-5 24477489 10.1007/s00415-013-7231-5 PMC4928702

[26] El Kossi M, Zakhary M. Oxidative stress in the context of acute cerebrovascular stroke. Stroke. 2000;31(8):1889–92. 10926952 10.1161/01.str.31.8.1889

[27] Turck N, Robin X, Walter N, Fouda C, Hainard A, Sztajzel R et al Blood Glutathione S-Transferase-pi as a Time Indicator of Stroke Onset. PloS one. 2012;7(9):e43830 23028472 10.1371/journal.pone.0043830 PMC3444482

[28] Akhtar M, Pillai K, Vohora D. Effect of thioperamide on oxidative stress markers in middle cerebral artery occlusion model of focal cerebral ischemia in rats. Hum Exp Toxicol 2008;27(10):761–7. 10.1177/0960327108094608 19042962 10.1177/0960327108094608

[29] Hacke W, Donnan G, Fieschi C, Kaste M, von Kummer R, Broderick J et al Association of outcome with early stroke treatment: pooled analysis of ATLANTIS, ECASS, and NINDS rt-PA stroke trials. The Lancet. 2004;363(9411):768–74. 10.1016/s0140-6736(04)15692-4 10.1016/S0140-6736(04)15692-4 15016487

[30] Marler J, Tilley B, Lu M, Brott T, Lyden P, Grotta J et al Early stroke treatment associated with better outcome: the NINDS rt-PA stroke study. Neurology. 2000;55(11):1649–55. 11113218 10.1212/wnl.55.11.1649

[31] Whiteley W, Tseng MC, Sandercock P. Blood biomarkers in the diagnosis of ischemic stroke: a systematic review. Stroke; a journal of cerebral circulation. 2008;39(10):2902–9. 10.1161/STROKEAHA.107.511261 18658039 10.1161/STROKEAHA.107.511261

[32] Ezkurdia I, Juan D, Rodriguez JM, Frankish A, Diekhans M, Harrow J et al Multiple evidence strands suggest that there may be as few as 19,000 human protein-coding genes. Human molecular genetics. 2014;23(22):5866–78. 10.1093/hmg/ddu309 24939910 10.1093/hmg/ddu309 PMC4204768

[33] Spratt NJ, Fernandez J, Chen M, Rewell S, Cox S, van Raay L et al Modification of the method of thread manufacture improves stroke induction rate and reduces mortality after thread-occlusion of the middle cerebral artery in young or aged rats. Journal of neuroscience methods. 2006;155(2):285–90. 10.1016/j.jneumeth.2006.01.020 16513179 10.1016/j.jneumeth.2006.01.020

[34] Longa EZ, Weinstein PR, Carlson S, Cummins MS. Reversible middle cerebral artery occlusion without craniectomy in rats. Stroke; a journal of cerebral circulation. 1989;20(1):84–91. 10.1161/01.str.20.1.84 2643202

[35] Petullo D, Masonic K, Lincoln C, Wibberley L, Teliska M, Yao D. Model development and behavioral assessment of focal cerebral ischemia in rats. Life Sciences. 1999;64(13):1099–108. 10210272 10.1016/s0024-3205(99)00038-7

[36] Gentleman R, Varey V, Dudoit S, Irizarry R, Huber W. Bioinformatics and Computational Biology Solutions Using R and Bioconductor. New York: Springer; 2005.

[37] Warnes GR, Bolker B, Lumley T. gplots: Various R programming tools for plotting data. R package version 2.6.0. http://www.inside-r.org/packages/gplots/versions/2-6-0.

[38] Durinck S, Spellman PT, Birney E, Huber W. Mapping identifiers for the integration of genomic datasets with the R/Bioconductor package biomaRt. Nature protocols. 2009;4(8):1184–91. 10.1038/nprot.2009.97 19617889 10.1038/nprot.2009.97 PMC3159387

[39] Lynch JR, Blessing R, White WD, Grocott HP, Newman MF, Laskowitz DT. Novel diagnostic test for acute stroke. Stroke; a journal of cerebral circulation. 2004;35(1):57–63. 10.1161/01.STR.0000105927.62344.4C 14671250 10.1161/01.STR.0000105927.62344.4C

[40] Adamski MG, Li Y, Wagner E, Yu H, Seales-Bailey C, Soper SA et al Expression profile based gene clusters for ischemic stroke detection. Genomics. 2014;104(3):163–9. 10.1016/j.ygeno.2014.08.004 25135788 10.1016/j.ygeno.2014.08.004 PMC4196244

[41] Laskowitz DT, Kasner SE, Saver J, Remmel KS, Jauch EC. Clinical usefulness of a biomarker-based diagnostic test for acute stroke: the Biomarker Rapid Assessment in Ischemic Injury (BRAIN) study. Stroke; a journal of cerebral circulation. 2009;40(1):77–85. 10.1161/STROKEAHA.108.516377 18948614 10.1161/STROKEAHA.108.516377

[42] Grond-Ginsbach C, Hummel M, Wiest T, Horstmann S, Pfleger K, Hergenhahn M et al Gene expression in human peripheral blood mononuclear cells upon acute ischemic stroke. Journal of neurology. 2008;255(5):723–31. 10.1007/s00415-008-0784-z 18465111 10.1007/s00415-008-0784-z

[43] Oh SH, Kim OJ, Shin DA, Song J, Yoo H, Kim YK et al Alteration of immunologic responses on peripheral blood in the acute phase of ischemic stroke: blood genomic profiling study. Journal of neuroimmunology. 2012;249(1–2):60–5. 10.1016/j.jneuroim.2012.04.005 22591946 10.1016/j.jneuroim.2012.04.005

[44] Barr TL, VanGilder R, Rellick S, Brooks SD, Doll DN, Lucke-Wold AN et al A genomic profile of the immune response to stroke with implications for stroke recovery. Biological research for nursing. 2015;17(3):248–56. 10.1177/1099800414546492 25124890 10.1177/1099800414546492

[45] Lamers KJB, Vos P, Verbeek MM, Rosmalen F, van Geel WJA, van Engelen BGM. Protein S-100B, neuron-specific enolase (NSE), myelin basic protein (MBP) and glial fibrillary acidic protein (GFAP) in cerebrospinal fluid (CSF) and blood of neurological patients. Brain Research Bulletin. 2003;61(3):261–4. 10.1016/s0361-9230(03)00089-3 12909296 10.1016/s0361-9230(03)00089-3

[46] Tang Y, Lu A, Aronow BJ, Sharp FR. Blood genomic responses differ after stroke, seizures, hypoglycemia, and hypoxia: Blood genomic fingerprints of disease. Annals of neurology. 2001;50(6):699–707. 10.1002/ana.10042 11761467 10.1002/ana.10042

[47] Denes A, Pinteaux E, Rothwell NJ, Allan SM. Interleukin-1 and stroke: biomarker, harbinger of damage, and therapeutic target. Cerebrovasc Dis. 2011;32(6):517–27. 10.1159/000332205 22104408 10.1159/000332205

[48] Banwell V, Sena ES, Macleod MR. Systematic review and stratified meta-analysis of the efficacy of interleukin-1 receptor antagonist in animal models of stroke. Journal of stroke and cerebrovascular diseases: the official journal of National Stroke Association. 2009;18(4):269–76. 10.1016/j.jstrokecerebrovasdis.2008.11.009 19560680 10.1016/j.jstrokecerebrovasdis.2008.11.009

[49] Toda N, Ayajiki K, Okamura T. Cerebral blood flow regulation by nitric oxide: recent advances. Pharmacological reviews. 2009;61(1):62–97. 10.1124/pr.108.000547 19293146 10.1124/pr.108.000547

[50] Cheng MY, Lee AG, Culbertson C, Sun G, Talati RK, Manley NC et al Prokineticin 2 is an endangering mediator of cerebral ischemic injury. Proceedings of the National Academy of Sciences of the United States of America. 2012;109(14):5475–80. 10.1073/pnas.1113363109 22431614 10.1073/pnas.1113363109 PMC3325724

[51] Dagonnier M, Cooke IR, Faou P, Sidon TK, Dewey HM, Donnan GA et al Discovery and Longitudinal Evaluation of Candidate Biomarkers for Ischaemic Stroke by Mass Spectrometry-Based Proteomics. Biomarker insights. 2017;12:1177271917749216. 10.1177/1177271917749216 29308009 10.1177/1177271917749216 PMC5751906

